# Engineering of Adhesion at Metal–Poly(lactic acid) Interfaces by Poly(dopamine): The Effect of the Annealing Temperature

**DOI:** 10.1021/acsapm.3c00672

**Published:** 2023-07-06

**Authors:** Georgios Kafkopoulos, Ezgi Karakurt, Ricardo P. Martinho, Joost Duvigneau, G. Julius Vancso

**Affiliations:** † Department of Materials Science and Technology (MTP) of Polymers and Sustainable Polymer Chemistry (SPC), University of Twente, Enschede 7522 NB, The Netherlands; ‡ Department of Molecules and Materials, MESA+ Institute for Nanotechnology, Faculty of Science and Technology, University of Twente, Enschede 7500 AE, The Netherlands

**Keywords:** polydopamine, thermal treatment, PLA, polymer−metal bonding, interfacial adhesion

## Abstract

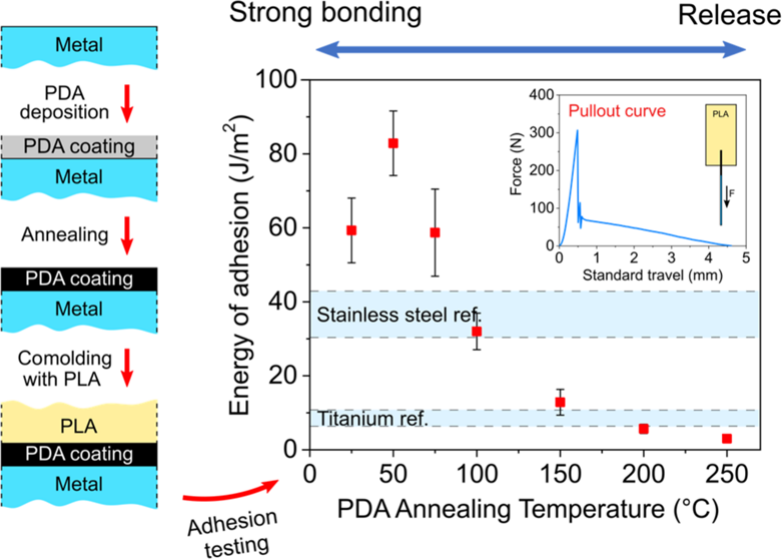

Control over adhesion at interfaces from strong bonding to release between thermoplastic polymers (TPs) and metal oxides is highly significant for polymer composites. In this work, we showcase a simple and inexpensive method to tune adhesion between a TP of growing interest, poly(lactic acid) (PLA), and two commercial metal alloys, based on titanium and stainless steel. This is realized by coating titanium and stainless steel wires with polydopamine (PDA), thermally treating them under vacuum at temperatures ranging from 25 to 250 °C, and then comolding them with PLA to form pullout specimens for adhesion tests. Pullout results indicate that PDA coatings treated at low temperatures up to a given threshold significantly improve adhesion between PLA and the metals. Conversely, at higher PDA annealing temperatures beyond the threshold, interfacial bonding gradually declines. The excellent control over interfacial adhesion is attributed to the thermally induced transformation of PDA. In this work, we show using thermogravimetric analysis, X-ray photoelectron spectroscopy, Fourier transform infrared, and ^13^C solid-state NMR that the extent of the thermal transformation is dependent on the annealing temperature. By selecting the annealing temperature, we vary the concentration of primary amine and hydroxyl groups in PDA, which influences adhesion at the metal/PLA interface. We believe that these findings contribute to optimizing and broadening the applications of PDA in composite materials.

## 1 Introduction

The production of thermoplastic composite (TPC) materials and structures is of paramount importance for applications that require lightweight materials with excellent mechanical performance.^1^ During processing, various interfaces form, for which interfacial adhesion plays a pivotal role in the overall material performance.^2^ Providing control over the adhesive bond strength at interfaces formed between thermoplastic polymers (TPs) and different classes of materials can be beneficial in many aspects. For instance, in TP–metal joints, strong bonding is required at the polymer–metal interface to transfer the load effectively.^3^ On the other hand, when considering processing methods of TPs that require easy release, e.g., injection molding, weak bonding at the polymer–metal interface where the polymer is in contact with the mold is needed.^4^ Even though a plethora of surface modification methods exist to affect adhesion at interfaces, they are often demanding and do not provide good control over a broad range of adhesion values.

Messersmith and co-workers^5^ introduced polydopamine (PDA) via the oxidative polymerization of dopamine in basic conditions and used it to coat a variety of substrates. The PDA polymerization process is simple and inexpensive^6^ and can be applied to coat virtually any material^7^ by dip- or spray-coating.^8^ The as-formed PDA layers are usually a few tenths of nanometers thick and are known to interact strongly with a broad range of substrates.^9,10^ In addition, PDA’s hydroxyl- and amine-rich chemical structure offers numerous possibilities for PDA to interact with other chemical groups.^11^ This renders PDA a strong candidate for connecting dissimilar materials forming strongly bonded interfaces.

By a simple treatment at elevated temperatures, thermal transformations that occur in PDA^12^ yield structural changes that allow one to tune interactions at interfaces. The thermal transformation of PDA has been the focus in a number of studies^12−21^ over the past decade. A simple thermal treatment at temperatures up to 150 °C is known to alter PDA’s chemistry by cyclization of primary amines,^12^ catechol oxidation,^15,17,20^ and crosslinking reactions.^13,18,21^ Such chemical transformations have been utilized to improve the mechanical properties of PDA coatings,^13,14^ as well as to tune the surface chemistry of PDA-coated materials.^15−20^ Despite the significance of PDA thermal treatments, the subject has received limited attention. The majority of reports focused either on a single^13,14,16−19,21^ or at most on three annealing temperatures.^12,15,20^ Lack of information limits our understanding of how PDA thermally transforms over a broad range of temperatures, potentially hindering the effective application of the coatings.

In the present work, we study the thermal transformation of PDA at temperatures ranging from 25 to 250 °C. The results allow us to choose the right temperatures to engineer adhesion between two commercial metals (alloys) and a thermoplastic matrix (TPM). The materials system used in this study consists of two commercially relevant metal alloys, based on titanium and stainless steel, and a TPM, i.e., poly(lactic acid) (PLA). PLA was chosen as a TPM due to its growing commercial importance which is due to its biodegradability and production from non-petrochemical sources.^22^ In addition, tunable interfacial adhesion at PLA–metal oxide interfaces is important for a number of applications, such as controlled release packaging, PLA–metal oxide nano-composites and PLA-metal tooling bonding during processing.^23,24^

In particular, PDA layers were formed on titanium and stainless steel wires, followed by thermal treatment under vacuum at temperatures ranging from 25 to 250 °C. Subsequently, unmodified and PDA-modified wires were comolded with PLA by compression molding to form pullout specimens. The specimens produced were then subjected to wire pullout tests to evaluate the interfacial energy of adhesion between PLA and the two metal alloys.

## 2 Experimental Section

### 2.1 Materials

Tris(hydroxymethyl)-aminomethane buffer (M: 121.14 g mol^–1^) and dopamine hydrochloride (M: 189.64 g mol^–1^) were purchased from Sigma-Aldrich (Zwijndrecht, the Netherlands) and were used as received. Toluene, acetone, and 2-propanol were obtained from VWR (Amsterdam, the Netherlands). PLA granules, Ingeo Biopolymer 4060D, were purchased from NatureWorks B.V. (Arendonk, Belgium). Titanium grade 5 (Ti6Al4V) wires with a diameter of 1 mm, a modulus (*E*_f_) of 110 GPa, and a surface roughness (*R*_a_) of 1.3 μm were obtained from SELFAN Fine + Metal GmbH (Köln, Germany). Stainless steel (SS-316) wires with a diameter of 1 mm, a modulus (*E*_f_) of 190 GPa, and a surface roughness (*R*_a_) of 1.1 μm were provided by Kuil Nicos (Enschede, the Netherlands). For more information and motivation on the surface morphology of the wires, please refer to the Supporting Information (SI-1).

### 2.2 Preparation and Cleaning of Metal Wires

The titanium wires were straightened using a tensile testing instrument (Zwick i-line Z5.0, Zwick/Roel, Ulm, Germany) with a force of 700 N for 10 min and afterward were cut into 9 cm long pieces. The stainless steel wires were not subjected to a straightening process. For both metal alloy wires, one tip of the 9 cm long pieces was hand-polished using 320-grit paper initially, followed by 800-grit sandpaper. The tip-polished wires were ultrasonically cleaned (2510 ultrasonic cleaner; Branson, Danbury, USA) using toluene, acetone, Milli-Q water (Milli-Q Advantage A10, Millipore), and 2-propanol for at least 30 min per solvent. The solvent-cleaned wires were dried at 200 °C under vacuum and stored in a sealed container. Hereinafter, wires subjected to the aforementioned process will be referred to as “unmodified wires”.

### 2.3 Coating of Metal Wires with PDA

**Figure 1:**
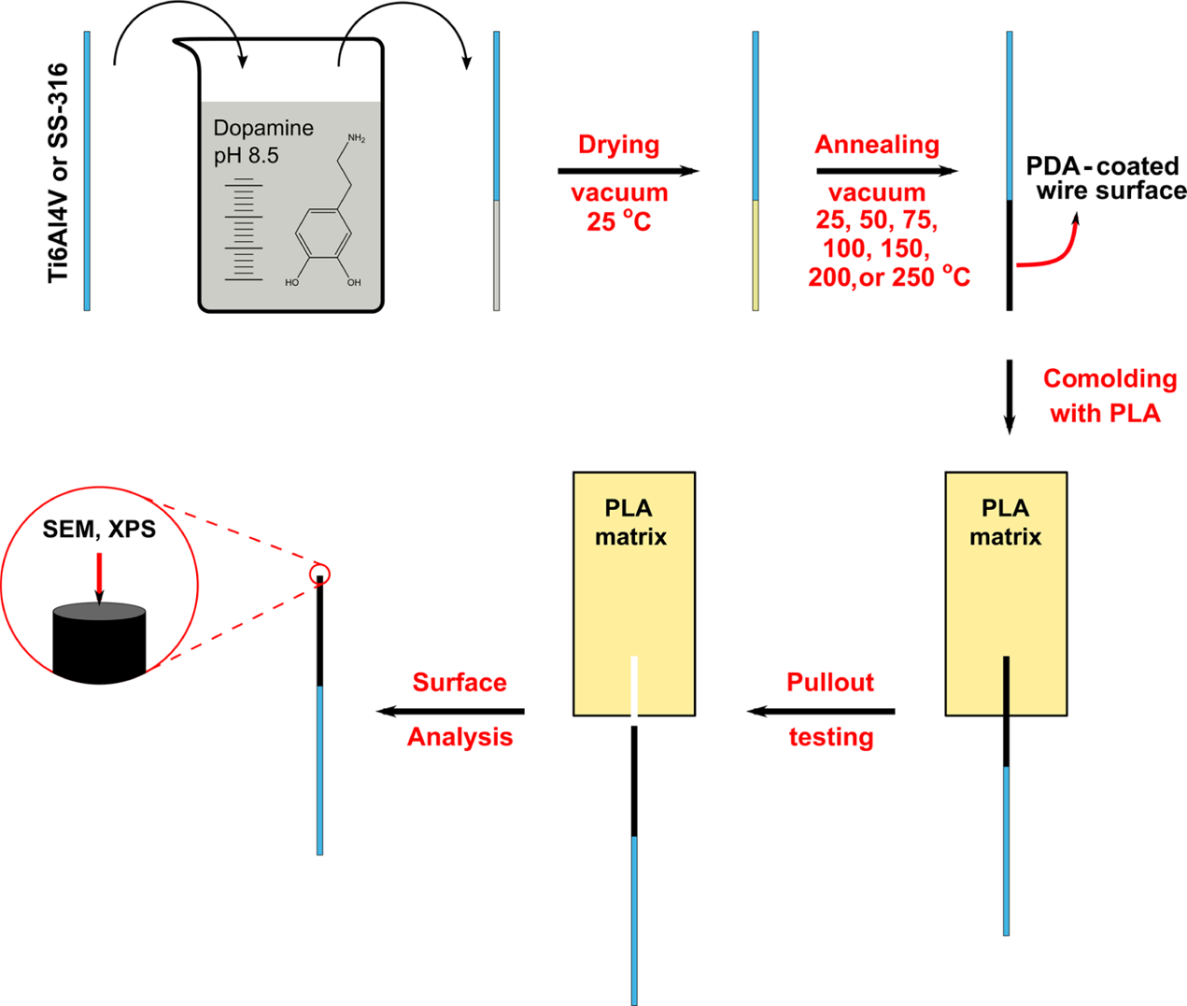
Schematic representation of titanium and stainless steel wire modification with polydopamine, PLA–metal wire comolding process, pullout testing, and fractured surface analysis.

In a typical PDA-coating process, eight unmodified titanium or stainless steel wires were placed in a plasma chamber (Plasma Prep II SPI; West Chester, USA) and treated with oxygen plasma at an oxygen pressure of 200 mtorr and a current of 40 mA for 1 min. After the O_2_ plasma treatment, the wires were placed directly in a 50 mL Erlenmeyer flask containing 50 mL of tris buffer (10 mM) solution with 5 mg mL^–1^ dopamine hydrochloride. The wires were left in the dopamine-tris buffer solution in ambient conditions for 24 h to form a PDA layer on their surface. Afterward, the PDA-coated wires were cleaned thoroughly with Milli-Q water and dried under vacuum at room temperature for 24 h. The coated wires will be referred to hereinafter as PDA@s.steel and PDA@titanium. Following the drying process, a group of PDA-coated wires were stored in ambient conditions, and the rest were annealed under vacuum at different temperatures, i.e., 50, 75, 100, 150, 200, or 250 °C. The PDA-coating and thermal treatment processes are schematically summarized in Figure 1.

### 2.4 PLA–Metal Wire Comolding and Pullout Processes

Metal wire–PLA comolded joints were produced by compression molding. PLA granules were dried at 60 °C for 4 h and were then comolded with the wires using a stainless steel mold. The mold design and details of the preparation process before compression molding are described in our previous work.^25^ Compression molding was performed using a THB 400 press (Fontijne, Delft, the Netherlands) at 180 °C with a pressure of 40 MPa for 5 min followed by a slow cooling down (∼30 min) to room temperature. The resulting pullout specimens consisted of a PLA block (20 × 40 × 4 mm^3^) in which approximately 5 mm of the metal wire was embedded. Prior to the pullout testing, the comolded specimens were stored under vacuum at room temperature for at least 24 h. Pullout testing was performed using a Zwick i-line Z5.0 (Zwick/Roel, Ulm, Germany) tensile testing apparatus at a cross-head speed of 10 mm min^–1^. Further details on the tensile grips are described in our previous work.^25^ At least 6 samples were tested for each sample group. The comolding process, pullout testing, and surface analysis of the embedded wires after pullout testing are schematically summarized in Figure 1.

### 2.5 Characterization Methods

Thermogravimetric analysis (TGA; TGA550, TA instruments) was employed to evaluate the thermal stability of PDA. PDA powders were obtained from the post-polymerization solution of PDA via five cycles of centrifugation (Z36HK, HERMLE Labotechnik, Germany) and Milli-Q washing, followed by drying under vacuum at 25 °C for 24 h. The resulting powder was then subjected to two modes of TGA measurements in a N_2_ atmosphere. The first was a temperature scan to evaluate the thermal stability of PDA and was performed from 25 to 600 °C at a rate of 10 °C min^–1^. The second was an isothermal measurement that aimed at evaluating the long-term stability and mass loss of PDA after exposure to different temperatures. Thus, PDA precipitates were heated up at a rate of 10 °C min^–1^ from 25 to 50, 75, 100, 150, 200, or 250 °C where they were kept isothermally for 180 min.

Mass spectrometry (QMS 403 D Aeolos MS, Netzsch) combined with TGA (STA 449 F3 Jupiler, Netzsch) (MS-TGA) was performed to identify the chemical species released during the thermal transformation of PDA. The measurement was performed on PDA powders (see previous paragraph) at a rate of 10 °C min^–1^ under a N_2_ atmosphere. The results are displayed in the Supporting Information (SI-6).

To provide insights into the effect of thermal treatments on the molecular structure of PDA, Fourier transform infrared (FTIR) spectroscopy and 13C solid-state NMR were employed. The measurements were performed on PDA powders obtained from the post-polymerization solution of PDA via five centrifugation (Z36HK, HERMLE Labotechnik, Germany) and Milli-Q washing cycles, followed by drying under vacuum for 24 h.

For FTIR measurements, PDA powders were annealed under vacuum at 50, 75, 100, 150, 200, or 250 °C and were then used to obtain FTIR–ATR (Alpha, Bruker, Leiderdorp, the Netherlands) spectra for each annealing temperature. The scanned spectrum ranged from 4000 to 400 cm^–1^ with a resolution of 4 cm^–1^ and was averaged over 64 scans. A 25-point smoothening was applied to the spectra, followed by a baseline correction. To identify the effect of thermal treatments at temperatures higher than 250 °C, post-test PDA powders from the TGA isothermal (25 to 600 °C) measurements were analyzed with FTIR using the aforementioned parameters. The FTIR results obtained from the TGA powders are displayed in the Supporting Information (SI-7).

Solid-state NMR experiments were performed with a Bruker 14.1 T (600.16 MHz for ^1^H) Avance Neo spectrometer equipped with a 2.5 mm magic angle spinning (MAS) probe. ^13^C cross-polarization (CP) MAS measurements were conducted at a spinning rate of 15 kHz, with a 90° pulse for ^1^H of 2.25 μs and a contact time of 2 ms and 14,336 averages. ^13^C spectra were externally referenced to adamantane and acquired with 2272 complex points with a spectral width of 301 ppm (45.5 kHz). All NMR spectra were zero-filled, apodized, subjected to 0th-order phasing, and Fourier-transformed using MestReNova.

Scanning electron microscopy (SEM; JSM 7610 FPlus, JEOL) was used to indicate the type of interfacial failure (adhesive/cohesive) that occurs during the pullout testing for each coating. The tip of the titanium and stainless steel wires (see Figure 1) was imaged using SEM before and after the pullout tests. The working distance (W.D.) and acceleration voltage were 11.4 mm and 1.5 kV, respectively. At least three wires were imaged for each sample group.

X-ray photoelectron spectroscopy (XPS; PHI Quantes, Physical Electronics) was performed on the tip of the wires (see Figure 1) at different stages of the preparation process and after pullout testing. The aim of the XPS measurements was (1) to ensure that the wires were completely covered with a PDA layer, (2) to identify the interfacial failure location after pullout testing with respect to the PDA layers, and (3) to quantify the surface atomic concentration of the PDA-coated wires after heat treatment at different temperatures. The measurements were performed using a monochromatic Al Ka source at 1486.6 eV with a beam diameter of 50 μm and an X-ray gun power of 12.5 W. The base pressure of the chamber was 7 × 10^–7^ Pa, and the working pressure was 1.3 × 10^–6^ Pa (argon). The beam input and detector input angles were 45°. The obtained XPS spectra and their respective analyses for determining the interfacial failure location are only shown in the Supporting Information (SI-3,5).

## 3 Results and Discussion

### 3.1 Testing Method and Analysis

**Figure 2:**
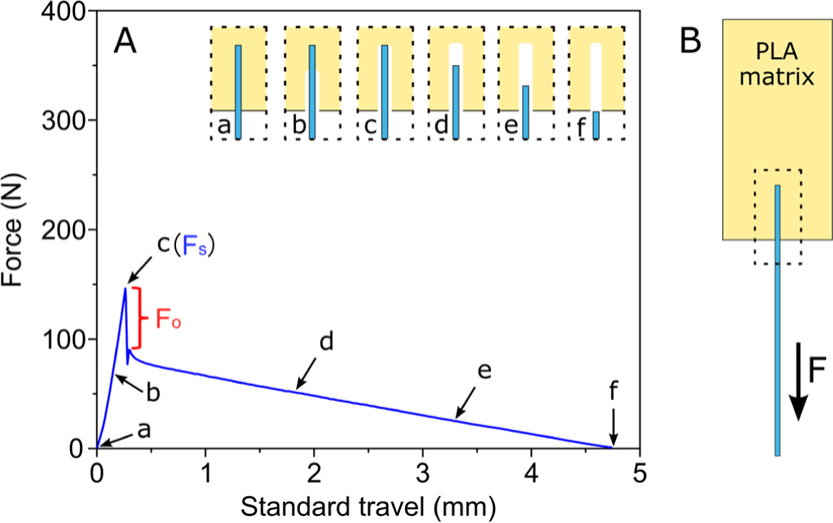
Typical pullout curve of a PLA/titanium wire pullout specimen (A). Schematic representation of a pullout specimen (B). The insets in “A” represent the state of the embedded wire at different stages of the pullout. “Standard travel” denotes the distance increase between the tensile cross-heads after the start of the pullout test.

Pullout tests were employed to evaluate the interfacial adhesion between PLA and the two metal alloys used in this study. Figure 2A shows a typical pullout curve obtained from a titanium wire embedded in a PLA matrix. Initially, the force increases linearly with the travel (points a–b–c in Figure 2A). In this part, the increment in the force causes a detachment of the PLA/titanium interface that progresses toward the embedded end of the wire. The debonding of the interface is completed at the slip point (point c in Figure 2A), after which a sudden drop in the force (*F*_o_) is observed, followed by a further decreasing trend with the standard travel. In this part of the curve, the wire is progressively pulled out of the PLA matrix until its complete removal while maintaining frictional contact at the titanium/PLA interface.

As mentioned in our previous work,^25,26^ the interfacial work of adhesion (*G*_a_) can be estimated by using the following equation:^27^
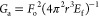
1where *r* is the radius, *E*_f_ is the wire’s modulus, and *F*_o_ is the debonding force in zero friction conditions.^28^ The value of *F*_o_ can be obtained from the pullout curves (indicated in Figure 2A), while the values of the diameter and modulus of the respective wires are known. Thus, with this methodology, we can quantify the interfacial work of adhesion between PLA and different metal alloys, as well as the effect of PDA coatings deposited on metal surfaces prior to the comolding process. The results in this work are discussed in terms of *G*_a_, while the *F*_o_ values are shown in the Supporting Information (SI-2).

### 3.2 Pullout Tests Using PDA Surface-Modified Titanium and Stainless Steel Wires

Pullout tests were employed to evaluate the effect of polydopamine layers on the interfacial adhesion of PLA–metal comolded joints. As-received and PDA-modified titanium and stainless steel wires were comolded with an amorphous PLA matrix, and the resulting specimens were subjected to pullout testing. The pullout curves were then analyzed based on the model described in Section 3.1 to obtain the interfacial work of adhesion (*G*_a_) values.

**Figure 3:**
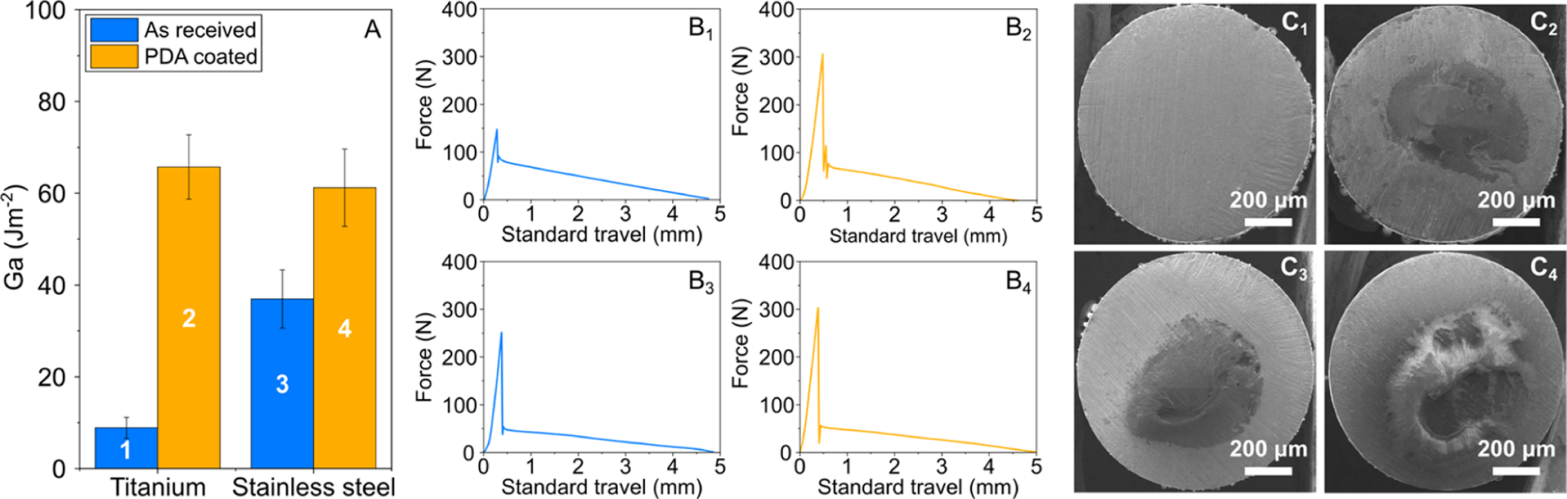
Energy of adhesion (*G*_a_) of PLA–metal comolded joints using unmodified and PDA-modified titanium and stainless steel wires (A). Typical pullout curves (B_1–4_) and SEM images of the wire tips after pullout testing (C_1–4_) for each type of metal/surface treatment are shown in plot A. The subscript numbers in B and C correspond to (1) unmodified titanium, (2) PDA-modified titanium, (3) unmodified stainless steel, and (4) PDA-modified stainless steel.

Figure 3A shows the calculated *G*_a_ values between PLA and the two metal alloys with and without a PDA coating on the metal surfaces. Typical pullout plots obtained from each sample group and used to estimate the *G*_a_ values can be seen in Figure 3B_1–4_. The pullout samples using unmodified titanium wires exhibited *G*_a_ values of approximately 9 J m^–2^, roughly 25% of that of unmodified stainless steel, i.e., 37 J m^–2^. Both titanium and stainless steel wires coated with a PDA nanolayer exhibited an enhanced bonding with PLA, with a similar *G*_a_ value of about 60 J m^–2^. The equally strong bonding between PLA and the two metal alloys, in the presence of a PDA layer, can be explained by the location of the interfacial failure. XPS measurements at the tip of the PDA-coated metal wires after pullout testing showed no traces of the metal substrate, indicating that the interfacial failure occurs at the PDA–PLA interfacial region. Details of the XPS experimental procedure and analysis can be found in the Supporting Information (SI-3). Thus, the PDA–metal interface is not the weakest link in both cases, resulting in similar adhesion values when using different metal substrates. The bonding strength of the aforementioned sample groups is also reflected in the failure mechanism at the interface. SEM images of the wire tips after the pullout testing (Figure 3C_1–4_) reveal that sample groups exhibiting low *G*_a_ values failed adhesively (Figure 3C_1_), while sample groups exhibiting high *G*_a_ values failed partly cohesively (Figure 3C_2–4_).

Overall, a PDA layer on the surface of either titanium or stainless steel results in an equally strong bonding with PLA. This is also supported by the observation that after pullout testing, interfacial failure occurs in the PDA–PLA interfacial region. This strongly indicates that the PDA layer bonds strongly with the metal substrate and is not the weak link of the joint even after processing at 200 °C.

### 3.3 Effect of PDA Thermal Pretreatment on the Adhesion with PLA

The effect of PDA thermal pretreatment on the interaction with the PLA matrix during the comolding process was also evaluated by pullout testing. PDA coatings resulted in similar *G*_a_ values regardless of using titanium or stainless steel wires (e.g., Figure 3A). PDA-coated stainless steel (and also titanium) wires were annealed at temperatures ranging from 25 to 250 °C, followed by comolding with a PLA matrix and then pullout testing.

**Figure 4:**
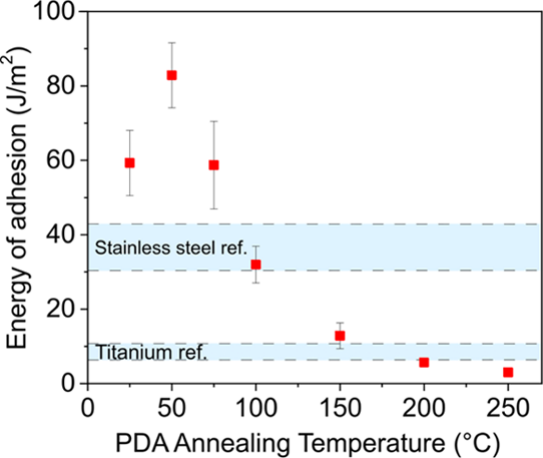
Energy of adhesion (*G*_a_) of PLA–PDA@s.steel wire comolded joints for different annealing temperatures of the PDA-coated stainless steel wires before the comolding process. The inset yellow stripes indicate the standard deviation of *G*_a_ values for as-received titanium and stainless steel samples (blue bars from Figure 4A).

**Figure 5:**
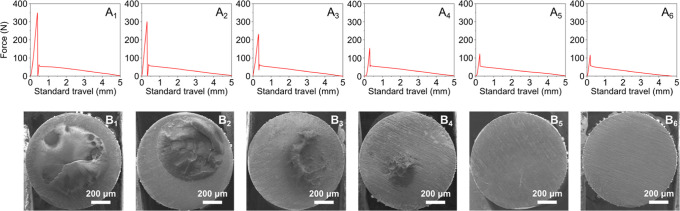
Representative pullout plots of PLA–PDA@s.steel comolded joints for different annealing temperatures of the PDA@s.steel wires before the comolding process (A_1–6_). SEM images of the wire tips after pullout testing (B_1–6_). The subscript numbers in A and B correspond to annealing temperatures of the PDA@s.steel wires of 50, 75, 100, 150, 200, and 250 °C, respectively.

Figure 4 shows the energy of adhesion (*G*_a_) of PLA–PDA@s.steel pullout samples at different annealing temperatures. Representative pullout curves for each data point are shown in Figure 5A_1–6_. By increasing the annealing temperature from 25 to 50 °C, a notable increase in *G*_a_ is observed from ∼60 to ∼80 J m^–2^. Above 50 °C, *G*_a_ follows a decreasing trend with the annealing temperature, resulting in a value of ∼4 J m^–2^ at 250 °C. SEM imaging was used to observe the failed interfaces. At low annealing temperatures, the SEM images show that cohesive failure is more pronounced with most of the wire tip surface being covered by PLA after pullout testing. By increasing the annealing temperature, increasing trends toward interfacial failure can be observed with no PLA matrix traces on the wire tip surface at 200 and 250 °C. Thus, the SEM observations support the failure energy trends observed. It is also noted that the interfacial failure location with respect to the PDA coatings remains the same as the one described in Section 3.2 (see Supporting Information SI-3), i.e., the PDA–metal interface is not the weakest link. Overall, applying a PDA layer followed by a simple thermal treatment appears to provide an excellent tool to tune adhesion between PLA and metal surfaces.

### 3.4 Effect of Thermal Treatment on the Chemical Structure of Polydopamine

Now we turn our attention to understanding why *G*_a_ shows such a strong dependence on the annealing temperature of the PDA. Given that the interfacial failure primarily occurs at the PDA/PLA interface, we focus on the interaction between PLA and PDA coatings following the comolding process. Hence, it is important to identify the changes induced in PDA when subjected to thermal treatments under vacuum. We note that atomic force microscopy (AFM) imaging of PDA-coated SiO_2_ wafers annealed at temperatures ranging from 25 to 250 °C revealed no significant changes in the roughness (*R*_a_) of the coatings (see Supporting Information SI-4). Thus, we assume that thermal treatments also do not affect the roughness of PDA coatings deposited on metal wires. This implies that we assign the dependence of *G*_a_ on the annealing temperature to molecular bonding mechanisms between PDA and PLA that we attempt to identify.

XPS measurements performed on the surface of annealed PDA-coated wires revealed that the O/C and N/C atomic ratios changed slightly from 0.27 to 0.29 for the O/C ratio and slightly decreased from 0.10 to 0.09 for the N/C ratio for annealing temperatures from 25 to 250 °C, respectively. However, these changes are within the statistical error. Significant changes in O/C and/or N/C ratios were observed only at annealing temperatures above 300 °C (see Supporting Information SI-5). The relatively unchanged N/C and O/C, between 25 and 250 °C, show that in this temperature range, the applied thermal treatments did not induce a notable change in the composition of the PDA coatings.

TGA, FTIR, and ^13^C solid-state NMR experiments were performed to gain further insights into the effect of thermal treatments on PDA. All measurements were performed on PDA powders obtained from the post-polymerization solution (see Section 2.5), which were thermally treated under vacuum.

TGA measurements were carried out using two different temperature profiles, i.e., dynamic and isothermal. Figure 7A shows the dynamic TGA curve of PDA obtained by a temperature scan from 25 to 600 °C at 10 °C min^–1^. In agreement with what has been previously reported,^13,29−32^ no sharp mass loss is observed with increasing temperature. Instead, PDA gradually loses mass upon increasing the temperature in what appears to be three temperature regimes with different mass loss slopes, i.e., ∼25–75 °C, ∼75–300 °C, and 300–600 °C. The mass loss between 25 and 75 °C has been previously assigned to the removal of physisorbed water.^13,33^ This is further supported in our work by isothermal TGA tests at 50 °C. The equilibrium mass loss at 50 °C is ∼9%, within the typical range for synthetic melanins,^34^ and is fully reversible between drying steps and exposure to atmospheric air (see Supporting Information SI-6). Based on this, we associate the mass loss between 25 and 75 °C with surface-bound water since the latter is known to be removed from melanins with mild thermal treatments at 60 °C.^35^ In the mass loss region between 75 and 300 °C, from the TGA isothermal curves shown in Figure 7, it is apparent that the equilibrium mass loss upon thermal treatment of PDA follows an increasing trend with increasing annealing temperature. These results suggest that at increasing annealing temperatures, a progressive alteration of the PDA chemical structure may take place. This then causes the increased release of species as a result of previously reported dehydration and other cross-linking reactions.^13^ It should be noted that MS–TGA measurements indicate both the removal of surface-bound water and the continuous removal of water from PDA at temperatures above 150 °C (see Supporting Information SI-6). Thus, for the temperature range between 75 and 300 °C, it is not possible to conclude whether the observed equilibrium mass losses correspond to the removal of “strongly” bound water^35^ in the PDA structure or that it is related to the product of dehydration and other crosslinking reactions. Finally, carbonization of PDA occurs at temperatures above 300 °C, as indicated by the O/C ratio trend (see Supporting Information SI-5) and as confirmed by previous reports.^36,37^

**Figure 6:**
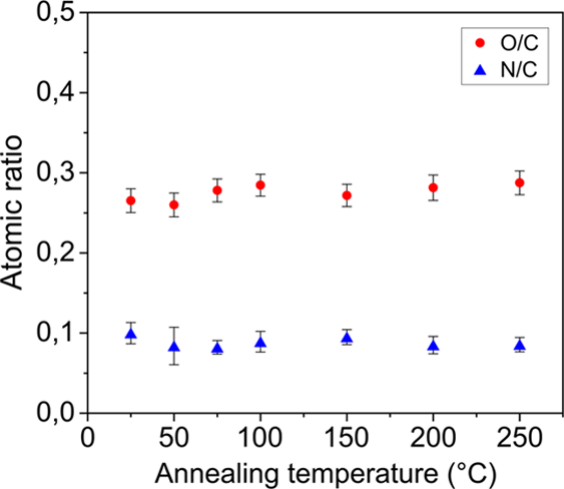
XPS determined N/C and O/C ratios of spectra obtained from the surface of PDA-coated metal wires subjected to thermal annealing under vacuum at temperatures ranging from 25 to 250 °C.

**Figure 7:**
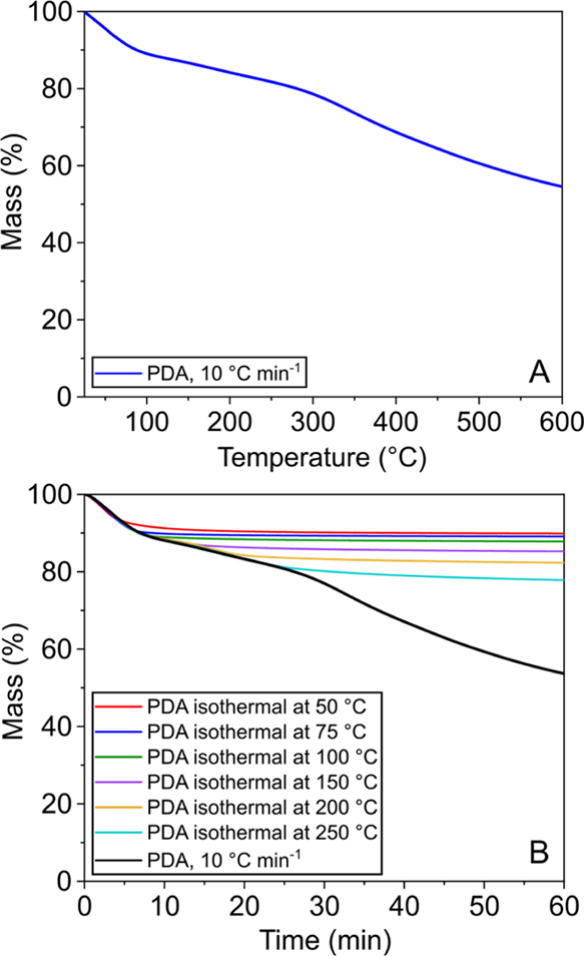
Dynamic (A) and isothermal (B) TGA curves of PDA powders.

**Figure 8:**
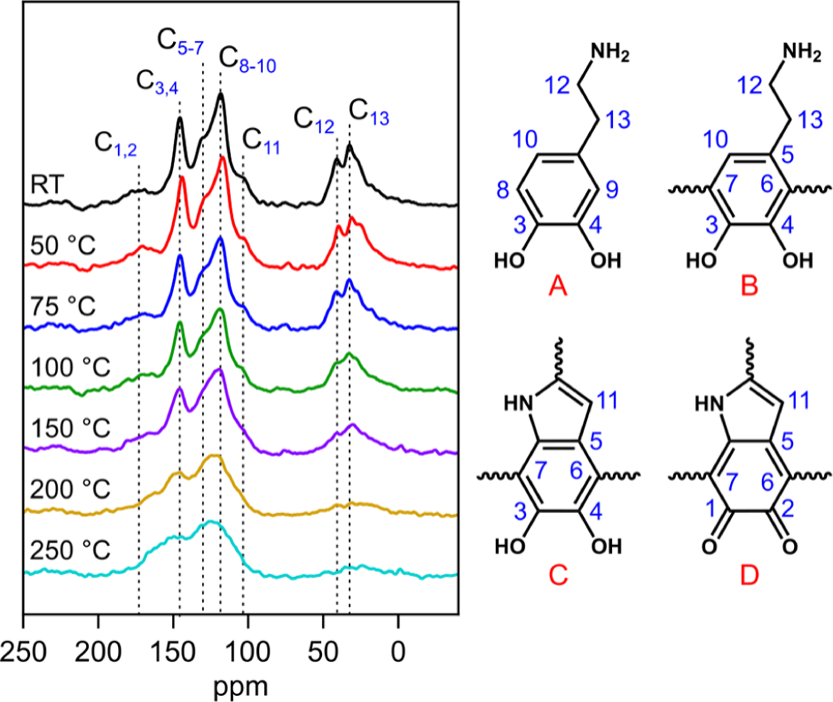
^13^C solid-state NMR spectra obtained from PDA powders annealed at temperatures from 25 to 250 °C. The chemical structures serve as indicative chemical species present in PDA: dopamine monomer (A), uncyclized dopamine (B), 5,6-dihydroxyindole (C), and 5,6-indoloquinone (D). The numbers 1–13 indicate the carbon species and their corresponding peaks are assigned in the NMR spectra.

Figure 8 shows the ^13^C solid-state NMR spectra of PDA powders annealed at temperatures ranging from 25 to 250 °C. Spectra were also obtained for annealing temperatures between 300 and 600 °C and can be found in the Supporting Information (SI-7). The peaks were assigned using the results of previous studies.^12,38,39^ We note that for simplicity, we assume that PDA consists of four main chemical species (structures A–D in Figure 8) that can be covalently and/or noncovalently bound. Needless to say, the structure of PDA is far more complex, with more reported building blocks^39^ and many features remaining unclear.^6^ However, the purpose of this measurement is to identify changes induced by thermal treatments and not to define the exact structure of PDA. From the evolution of the ^13^C solid-state NMR spectra by increasing the annealing temperature, two main trends can be observed. The first is associated with the peaks located at 30–40 ppm, which are assigned to the aliphatic carbons of uncyclized dopamine species. By increasing the annealing temperature, a decreasing trend is observed in the intensity of the peaks assigned to C_12_ and C_13_ carbons (pointed in Figure 8). Based on the relatively unchanged N/C ratio after thermal treatment of PDA (see Figure 6), we interpret this trend as the previously reported cyclization of primary amines at elevated temperatures.^12^ This cyclization process is thought to be completed at annealing temperatures above 150 °C as the peaks located at 30–40 ppm essentially disappear at annealing temperatures above 150 °C. The second trend is observed in the aromatic region, i.e., 100–170 ppm, where all the peaks tend to broaden by increasing the annealing temperature. NMR linewidths are proportional to correlation time and thus molecular weight.^40^ This observation indicates that further polymerization of monomeric, polymeric, and oligomeric species may take place. The further polymerization of PDA at elevated temperatures is also supported by previous reports and has been considered to improve its mechanical properties.^13^ The overall decrease in intensity of the spectrum can also be associated with the reduction in hydrogen content as cross-polarization experiments derived their ^13^C intensity from the hydrogen polarization.

**Figure 9:**
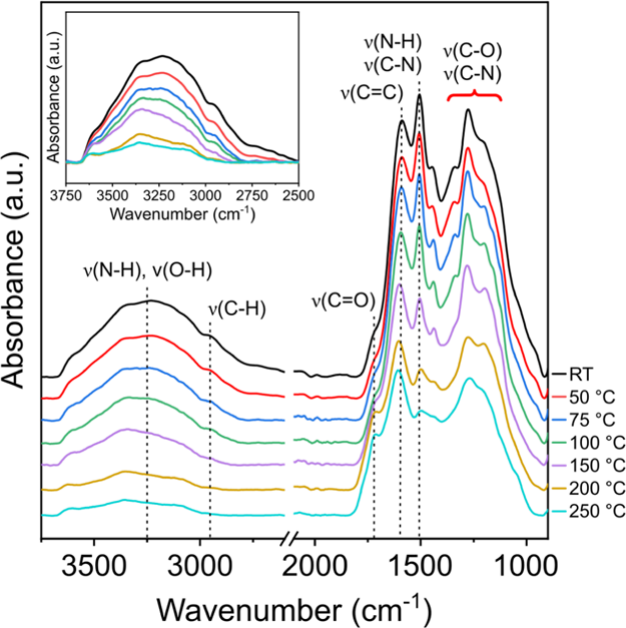
FTIR spectra of PDA powders annealed at temperatures ranging from 25 to 250 °C.

Figure 9 shows the FTIR spectra obtained from PDA powders annealed from 25 to 250 °C. The broad band located at ∼3300 cm^–1^ is associated with the ν(N–H) and ν(O–H) stretching vibrations originating from primary amines, water, and catechol groups.^41−43^ The “shoulders” at ∼2950 and ∼1720 cm^–1^ correspond to the ν(C–H) stretching of aliphatic carbons^19,43,44^ and ν(C=O) vibrations of quinone groups,^43,45^ respectively. The peak at ∼1585 cm^–1^ is assigned to the ν(C=C) stretching mode of the aromatic rings.^43,46^ The peak at ∼1505 cm^–1^ has been previously attributed to the ν(C–N) stretching^47−49^ and ν(N–H) scissoring^19,47^ vibrations. Finally, the overlapping peaks between ∼1405 and ∼1060 cm^–1^ are assigned to ν(C–O)^46,50,51^ and ν(C–N)^52^ vibrations.

By increasing the annealing temperature, the broad peak located at ∼3300 cm^–1^ follows a decreasing trend in absorption intensity. This indicates that (1) the removal of water from PDA, observed with MS–TGA and TGA, takes place; (2) the reduction of primary amine content, as interpreted by ^13^C solid-state NMR and proposed by previous studies,^17,20^ occurs; and (3) the oxidation of catechol groups to quinones, also reported by numerous studies, also occurs.^15−18^ The oxidation of the catechol groups is also supported by the increase in the quinone content, indicated by the increase in the shoulder peak located at ∼1720 cm^–1^ at elevated temperatures. Furthermore, by increasing the annealing temperature, the intensity of the ν(C=C) peak at ∼1585 cm^–1^ remains relatively unchanged, while the peak at ∼1505 cm^–1^ decreases. The peak at ∼1505 cm^–1^ is within the fingerprint region of the IR spectrum where a significant overlapping of peaks occurs, originating from other, yet unidentified, vibrations. The intensity decrease is assigned to the reduced contribution of the ν(N–H) scissoring vibrations due to a decrease in the primary amine content; however, the peak overlapping restricts clear interpretation.

Overall, for annealing temperatures between 25 and 250 °C, we propose that the following changes occur in PDA. At relatively low temperatures, i.e., 50 °C, the only observed change is the removal of surface-bound water. At higher temperatures, a continuous removal of water indicates either the presence of “strongly” bound water^35^ or the occurrence of dehydration reactions. With respect to the PDA structure, between 75 and 250 °C, three main changes are observed: the oxidation of catechols to quinones, the cyclization of primary amines, and crosslinking reactions. The above interpretations are in agreement with previous reports;^12−21^ however, here, we also clearly show that the equilibrium thermal transformation of PDA is directly connected to the annealing temperature. This means that the higher the annealing temperature, the lower the concentration of primary amine and catechol groups, the higher the concentration of quinones and the higher the degree of cross-linking in the PDA structure.

The interpretation of the thermally induced chemical transformation of PDA was performed under the assumption that PDA films and PDA powders comprise the same building blocks. This denotes that films and powders follow similar thermally induced molecular transformation paths. Hence, this assumption would allow us to combine the XPS measurements performed on PDA-coated wires and TGA and ^13^C solid-state NMR measurements performed on PDA powders to identify the changes induced in PDA by thermal treatments. It should, however, be noted that variations between PDA films and PDA precipitates have been previously reported.^53^ The particular study using MALDI-ToF concluded, within the limitations associated with this methodology, that PDA precipitates and films share the main chemical component (*m*/*z* 402) but differ in minor species which are present only in PDA precipitates. In the present work, FTIR spectra obtained from PDA powders and ∼70 nm PDA films deposited on SiO_2_ wafers (see Supporting Information SI-7) exhibited no significant deviations. In addition, observed changes in thermally treated PDA powders, e.g., the oxidation of catechols to quinones, were also in agreement with studies on PDA films.^15−18^ Based on the above, we propose that even though PDA powders likely exhibit some differences from PDA films, they may still provide valuable insights that could be correlated with the chemical changes thermal treatments induce in PDA films.

### 3.5 Proposed PDA–PLA Bonding Mechanism

Section 3.4 provides the chemical basis for understanding the dependence of *G*_a_ values on the PDA annealing temperature, as shown in Figure 4. Annealing temperatures of 50 °C cause the removal of surface-bound water, as indicated by TGA and MS–TGA measurements. Water is known to cause PLA chain scission at elevated temperatures by hydrolysis,^54^ and we note that the pullout samples are comolded at 180 °C. Thus, we associate the observed increase in *G*_a_ from 25 to 50 °C with the absence of PLA hydrolysis from water bound on the surface of PDA.

**Figure 10:**
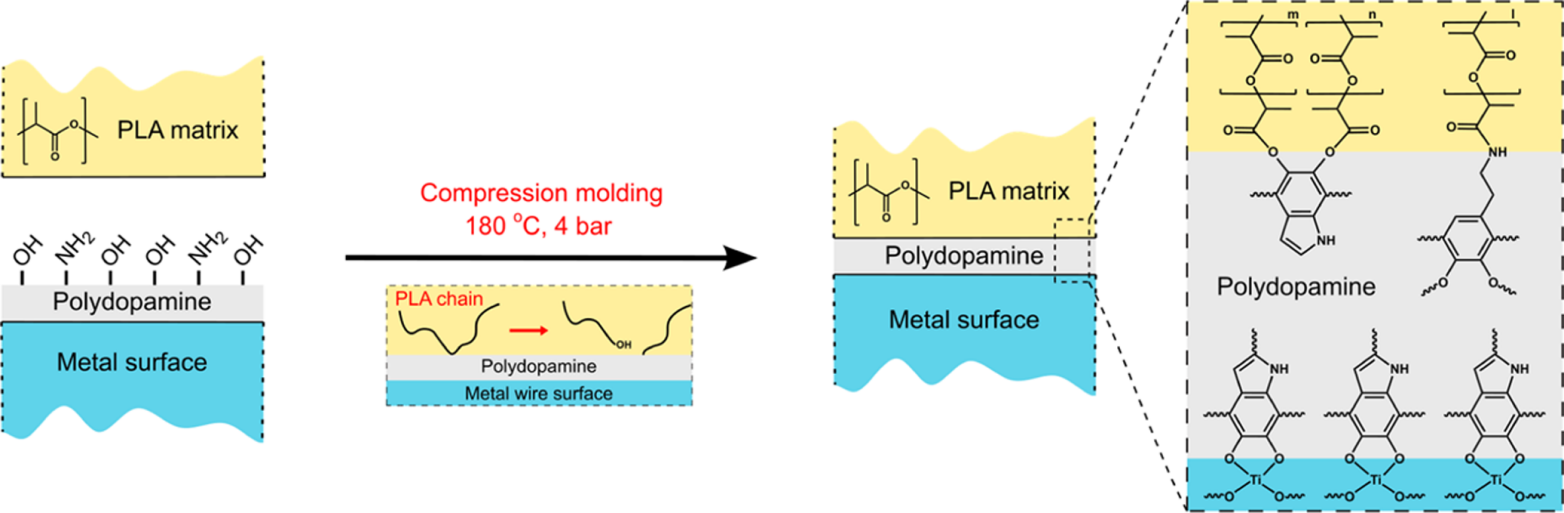
Proposed bonding mechanism between a polydopamine coating and the PLA matrix during the comolding process of pullout test specimens.

At annealing temperatures between 75 and 250 °C, a progressive oxidation of catechols to quinones and cyclization of primary amines is proposed. In the same temperature range, the *G*_a_ values follow a decreasing trend, which indicates that *G*_a_ is associated with the interaction of primary amine and hydroxyl groups present in PDA coating with the PLA matrix. For reasons that will be apparent further below, the interaction between PLA and PDA is assigned to the formation of covalent bonds during the comolding process. The most probable covalent bond formation mechanisms are transesterification and aminolysis reactions, known to occur in PLA melts, between the ester groups of PLA and hydroxyl^55^ or primary amine^56^ groups, respectively. Such bonding mechanisms would result in a PLA chain scission at the PLA/PDA interface, with shorter PLA chains attached to the PDA coating. This is expected to result in more PLA chains to covalently attach to the PDA film, hence enhancing bonding at the PDA/PLA interface. The proposed bonding mechanism is schematically represented in Figure 10.

At this point, a note on the thermal transformation of PDA within the context of this work should be made. The comolding process requires processing temperatures of 180 °C; hence, the PDA coatings are also expected to thermally transform during the joining process. This has two implications. First, an improvement in the mechanical properties of the coatings, previously reported for thermally annealing PDA,^13,14^ takes place, which is likely a major contributing factor to their applicability as adhesive interlayers. Second, the primary amine and hydroxyl group concentration declines, which introduces a competition at the PDA surface between reactions with the PLA matrix and the thermal transformation process of PDA. Thus, for PDA to perform well as an adhesive interlayer between titanium and PLA during the joining process, the exposure time of PDA coatings to high temperatures before being in contact with the PLA melt needs to be minimized.

The thermal transformation of PDA during the comolding process also provides the basis for the hypothesis of covalent bond formation between PLA and PDA. There are two possible forms of interaction between PDA and PLA, i.e., covalent bonds and secondary interactions. If secondary interactions were the ones to regulate the interaction between PDA and PLA, then the *G*_a_ values should be similar for annealing temperatures between 25 and 150 °C. However, in this temperature range, a significant variation of *G*_a_ is observed. Hence, even though secondary interactions could still contribute to adhesion, they do not appear to be a dominant factor, which leads us to hypothesize that the interfacial bonding between PDA and PLA is mainly governed by the formation of covalent bonds.

### 3.6 Applications and Perspectives

PDA’s application and thermal transformation were showcased as a simple, inexpensive yet effective tool to modify and tune the surface functionality of metals. By thermally annealing PDA and thus changing the surface concentration of primary amine and hydroxyl groups, control over adhesion in PLA–metal comolded joints was achieved. This highlights the potential of PDA coatings for a broad range of composite applications. PDA coatings may be used in polymer/metal hybrid joints as a primer to maximize the interfacial bond strength.^57^ This can be realized more effectively using fast PDA deposition techniques such as electro-spraying^58^ or PDA spray deposition combined with oxidizing agents.^8^ In polymer processing, there are cases where adhesion between thermoplastics and metal tooling is undesired, e.g., mold–polymer interaction in injection molding. There, the thermal treatment of PDA can be of benefit since it may not only reduce the coating–polymer bonding but would also result in mechanically more robust coatings that are able to survive many processing cycles. Such an application may be rendered competitive by using advanced techniques to anneal PDA coatings. An example is laser annealing which has been recently showcased to anneal PDA, resulting in coatings with a scratch resistance better than that of inorganic substrates.^59^

PDA coatings are particularly attractive due to the strong bonding they offer with a broad range of substrates by utilizing a wealth of molecular mechanisms.^9,10^ This has contributed to the application of such coatings to bridge interfaces beyond the ones studied in this work, i.e., polymer–metal. PDA has also been implemented in polymer composite materials as fiber sizing,^60^ nanofiller modification,^24^ or even nanofiller in the form of PDA nanoparticles.^61^ The incorporation of PDA in such systems, apart from promoting interfacial adhesion, also utilized other beneficial properties provided by PDA, such as UV resistance^62^ and flame retardancy.^63^ A deeper understanding of PDA chemistry and its bonding mechanisms with thermoplastic polymers may provide tools to engineer interfaces for optimizing the performance of polymer composite materials.

## 4 Conclusions

In this work, the thermal transformation of PDA has been exploited to provide control over adhesion between a TPM and two types of metal alloys. In particular, stainless steel and titanium wires with and without a PDA layer on their surface were comolded with PLA. The joints obtained were then subjected to pullout testing, revealing that a PDA layer is capable of greatly increasing interfacial bonding. To provide control over adhesion, PDA-coated wires were subjected to thermal treatment under vacuum prior to the comolding process with PLA at temperatures ranging from 50 to 250 °C. A thermal pretreatment at 50 °C promoted a further increase in the interfacial adhesion. This was assigned to the removal of surface-adsorbed water on the PDA layer, thus potentially preventing the hydrolysis of PLA chains during the joining process. Annealing temperatures above 50 °C resulted in a gradual decremental trend of the interfacial energy of adhesion, from ∼85 J m^–2^ at 50 °C to ∼3 J m^–2^ at 250 °C. We propose that the control over adhesion provided by a simple thermal treatment of PDA results from the unique and well-defined thermal transformation of PDA, which allows tuning the surface concentration of reactive chemical groups. Overall, we have demonstrated that a simple thermal pretreatment of PDA may tune the interfacial adhesion between two metal alloys and PLA ranging from strong bonding to release. This simple and inexpensive methodology shows great potential for applications ranging from TPM–metal composite materials and structures that require strong interfacial adhesion to release applications in TPC processing.

## Supporting Information Available

Surface morphology of metal wires (SEM, optical microscopy); *F*_o_ values of pullout tests; interfacial failure location analysis; surface morphology (AFM) and atomic concentration (XPS) of annealed PDA coatings; PDA water absorption (TGA, MS-TGA); solid-state NMR and FTIR curves of annealed PDA powders up to 600 °C; and FTIR spectra comparison between PDA powders and films (PDF)
