# Supplementary material for: Engineering of Adhesion at Metal–Poly(lactic acid) Interfaces by Poly(dopamine): The Effect of the Annealing Temperature
Source: ACS Appl Polym Mater. 2023 Jul 6;5(7):5370–80. doi: 10.1021/acsapm.3c00672 (PMC10353006; doi:10.1021/acsapm.3c00672)
Supplement: Supplementary file 1 — ap3c00672_si_001.pdf [file ap3c00672_si_001.pdf]

Supporting Information

Engineering of adhesion at metal-poly(lactic acid) interfaces by poly(dopamine): The effect of the annealing temperature

Georgios Kafkopoulos,[†] Ezgi Karakurt,[†] Ricardo P. Martinho,[§] Joost Duvigneau[†] and Julius G. Vancso*[,†]

[†]Department of Materials Science and Technology (MTP) of Polymers and Sustainable Polymer Chemistry (SPC), University of Twente, Enschede 7522 NB, the Netherlands.

[§]Department of Molecules and Materials, MESA+ Institute for Nanotechnology, Faculty of Science and Technology, University of Twente, 7500 AE Enschede, The Netherlands

*Corresponding Author
*E-mail: g.j.vancso@utwente.nl (G.J.V.).

SI-1. Titanium and stainless steel surface morphology

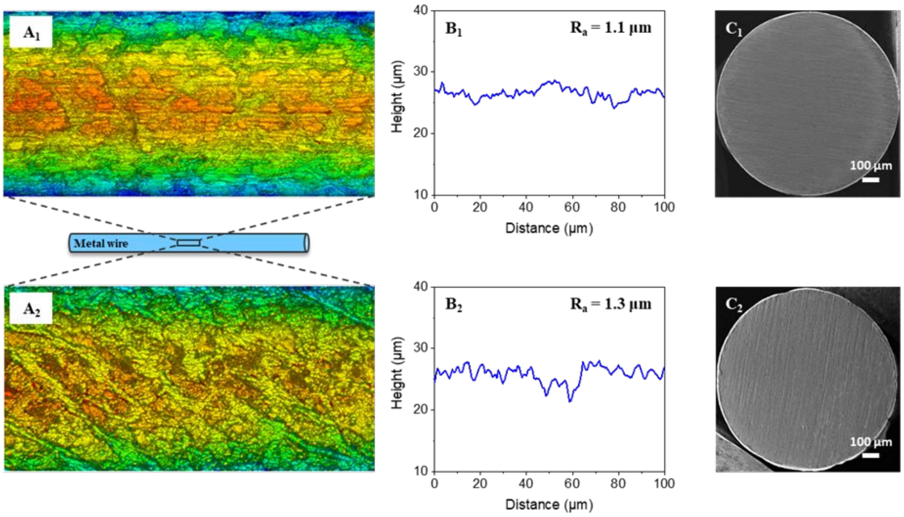

Figure S$_1$. Confocal microscopy images of the SS-316 wire surface (A_1) and Ti6Al4V wire surface (A_2); the scan area for both images is 500 x 250 µm. Example of the linear roughness profile of the SS-316 wire surface (B_1) and Ti6Al4V wire surface (B_2), extracted from A_1 and A_2, respectively. SEM image of the polished tip of the Ti6Al4V (C_1) and SS-316 wire surface (C_2).

Figure S2 provides an indication of the titanium and stainless steel wires surface morphology. The choice of the particular surface roughness (rough instead of smooth surface) was made for technical reasons related to the geometry and processing required to produce our test specimens (pullout). When smooth wires were used, we report that complete debonding at the PLA-metal interface occurred due to thermal stresses that are developed during processing. Thus, when using smooth wires it is not possible to obtain a reference value, and by extent a comparative measurement in the presence of PDA would not be feasible. On the other hand, roughness values of approximately 1 µm provided a window to both obtain a reference value and accurately measure the maximum adhesion value achieved in this work.

SI-2. Fo values defined from pullout tests

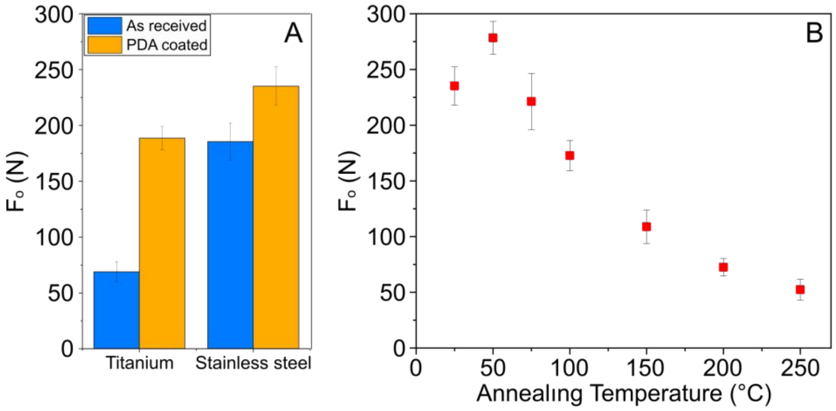

Figure S$_3$. F_o values were determined from pullout plots used to calculate the G_a values shown in Figure 4A (A) and Figure 5 (B).

SI-3. Identification of the interfacial failure location

XPS spectra were obtained from the tip of the clean titanium and stainless steel metal wires (Figure S3 A). The spectra indicate the presence of elements each alloy consists of, i.e. Ti, Al, O for titanium and Fe, Cr and O for stainless steel, as well as some atmospheric carbon contamination that is known to be present on the surface of metals exposed to atmospheric air.[1] After subjecting the metal wires to the PDA coating process, the only elements detectible by XPS are C, N, and O (Figure S3 B). The XPS spectra have a maximum information depth of 9-10 nm (Al Ka source),[1] thus we conclude that the wires are fully coated with a PDA layer of an average thickness greater than 10 nm.

XPS measurements were also performed on the tip of clean and PDA-coated metal wires after pullout testing from the PLA matrix. It should be noted that all the XPS spectra of the metal wires after pullout testing were obtained from locations where cohesive failure was not observable with SEM imaging. Examples of such locations are noted with red dots in the inset SEM images present in Figure S3 C and D. For clean wires, the XPS spectra (Figure S3 C) after pullout testing reveal that the elements originating from both of the respective metal alloys (see Figure S3 A) are still detectible. However, in the case of the PDA-coated wires no elements present in the respective metal alloys can be traced and the only detectible elements are C, N, and O (Figure S3 D). This indicates that after pullout testing, the PDA layer remains on the surface of the metal wires, with no part of the metal surface to be exposed with respect to the maximum information depth of 9-10 nm of the source used for the XPS measurements.

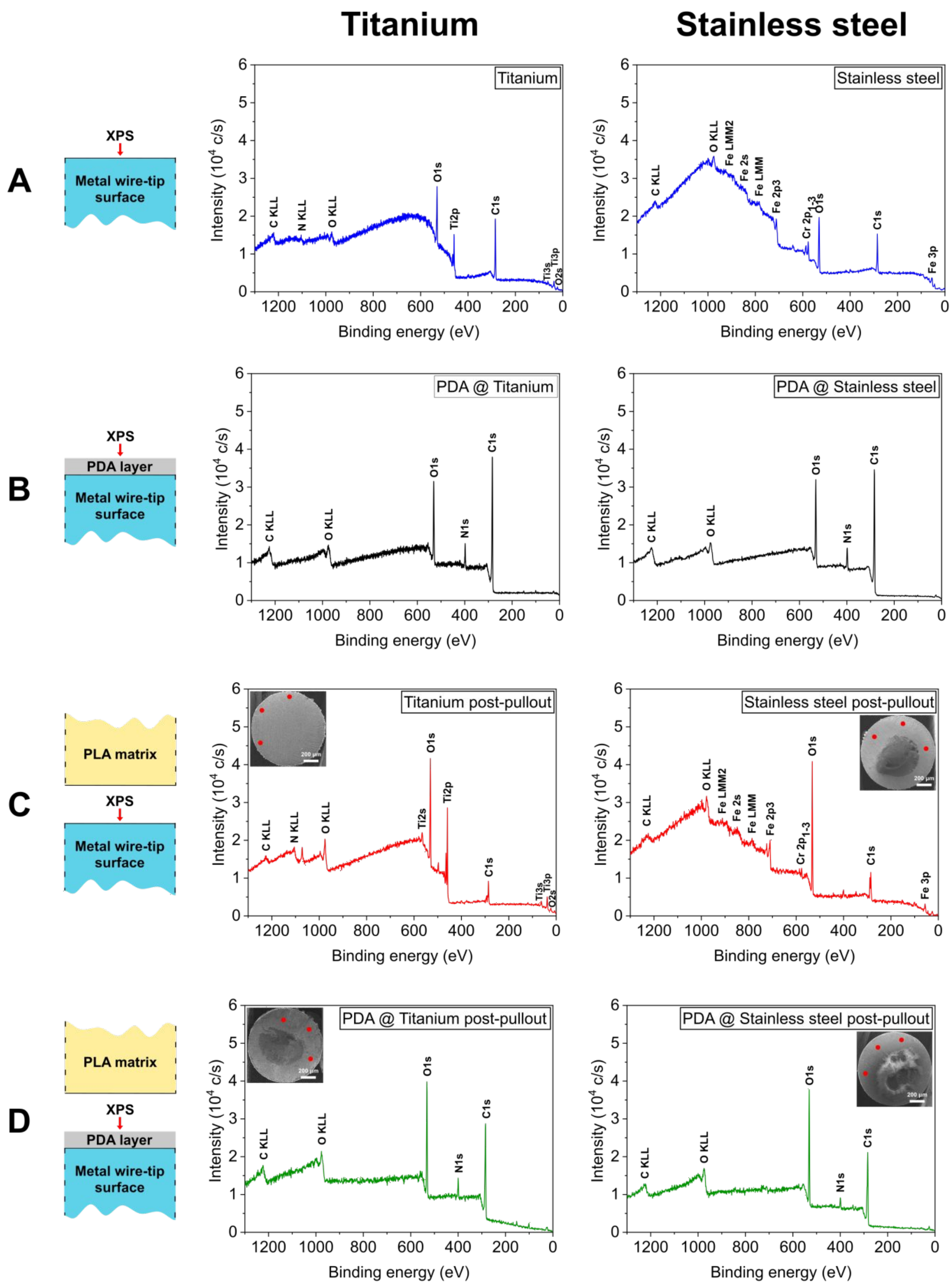

Figure S₄. XPS spectra obtained from the tip of titanium and stainless steel wires at different stages (A-D) of the sample preparation and testing processes: Unmodified metal wire (A), PDA coated metal wire (B), unmodified metal wire after pullout testing from a PLA matrix (C) and PDA coated metal wire after pullout testing from a PLA matrix (D). The inset SEM images in stages C and D are shown to point indicative location points (red dots) where the XPS spectra are obtained.

In addition, the N/C and O/C elemental ratios of the PDA-coated metal wires before and after pullout testing were calculated from the XPS spectra and are shown in table S1. The N/C ratio is reduced from 0.10 and 0.11 before to 0.09 and 0.08 after pullout testing for titanium and stainless steel, respectively. On the contrary, the O/C ratio is increased from 0.25 and 0.29 before to 0.43 and 0.52 after pullout testing for titanium and stainless steel, respectively. The reduction of N/C ratio and the increase of the O/C ratio can be accounted to the presence of PLA, which remains on the surface of the PDA layer after the pullout testing due to the covalent bonding formed during the co-molding process. This is further supported by the C/O ratios observed for the post-pullout PDA coated wires, which were found to be between the theoretical ratio for PLA and the measured ratio before pullout testing. Overall, based on the fact that no elements originating from the metal alloys were detected after pullout testing for PDA-coated wires, in combination with the observed changes in the O/C ratios we conclude that interfacial failure statistically occurs at, or at least close to, the PDA-PLA interface.

Table S_1: Theoretical and XPS determined N/C and O/C elemental ratio of PLA, PDA, and post-pullout test metal surfaces.

	**PLA (Theoretical)**	**PDA (Theoretical)**	**PDA@Ti (XPS)**	**PDA@Steel (XPS)**	**PDA@Ti post pullout (XPS)**	**PDA@steel Post pullout (XPS)**
**N/C**	0	0.125	0.10	0.11	0.09	0.08
**O/C**	0.67	0.25	0.27	0.29	0.43	0.52

SI-4. Surface morphology of annealed PDA coatings

PDA coatings of ~70 nm were deposited on SiO_2 wafers (see Figure S5A), which were then subjected to thermal treatments ranging from 25 °C to 250 °C. Atomic force microscopy (AFM, MultiMode 8, HarmoniX, NanoScope V controller, JV vertical engage scanner, Bruker, Santa Barbara, USA) was employed to evaluate the effect of thermal treatments on the surface morphology of PDA coatings. The samples were imaged in Peak Force Quantitative Nanomechanical Mapping mode (PF-QNM) using a Tap150Al-G silicon AFM probe with a nominal spring constant of 5 Nm^{-1}, a nominal tip radius of 10 nm and a nominal resonance frequency of 150 kHz. The thermal tuning method was used to calculate the AFM optical sensitivity and NanoScope Analysis software (version 1.9) was used to process the obtained data further.

The height map AFM images of PDA coatings annealed between 25 °C and 250 °C are shown in Figure S5B. The average R_a values shown in Table S_2, range from 9.9 to 15.2 nm with no observed trends by increasing the annealing temperature. Based on this, we conclude that the surface morphology of polydopamine coatings is not impacted, at least not significantly, at annealing temperatures up to 250 °C. Thus we assume that the dependence of G_a values on the annealing temperature (please refer to section 3.3 of the main article) is not related to the surface morphology of the coatings.

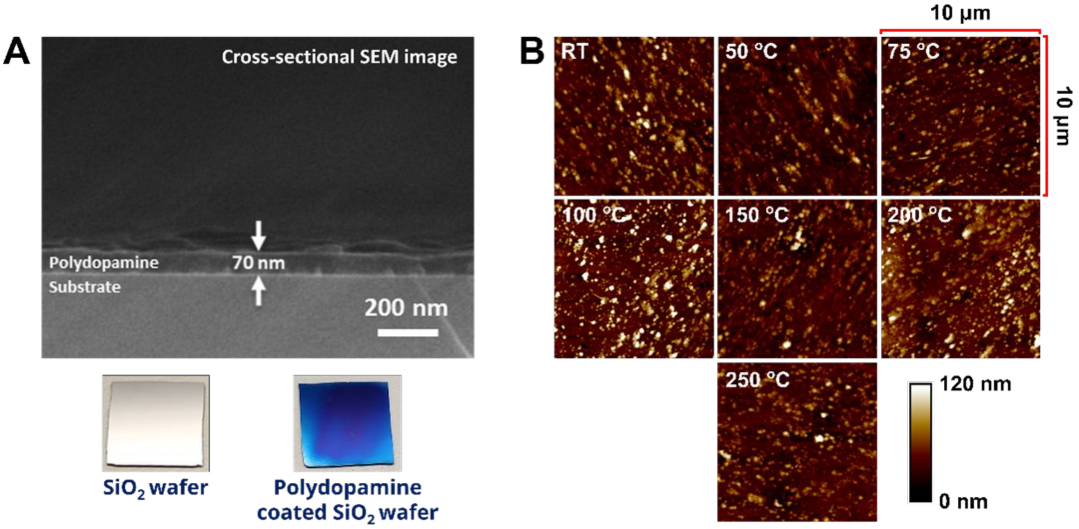

Figure S5. Photographs of SiO_2 silicon wafers before & after coating with PDA and cross-sectional SEM image of SiO_2 wafer coated with a ~70 nm PDA layer (A). AFM height maps of PDA layers on SiO_2 wafers annealed at temperatures ranging from 20 °C to 250 °C.

Table S2. Roughness (R_a) values of thermally annealed PDA coatings were calculated from the AFM height map images shown in Figure S5B.

**Annealing temperature**	**RT**	**50 °C**	**75 °C**	**100 °C**	**150 °C**	**200 °C**	**250 °C**
**R_a (nm)**	12.1	9.9	10.3	15.2	10.4	13.7	10.6

SI-5. Atomic concentration of annealed polydopamine coatings

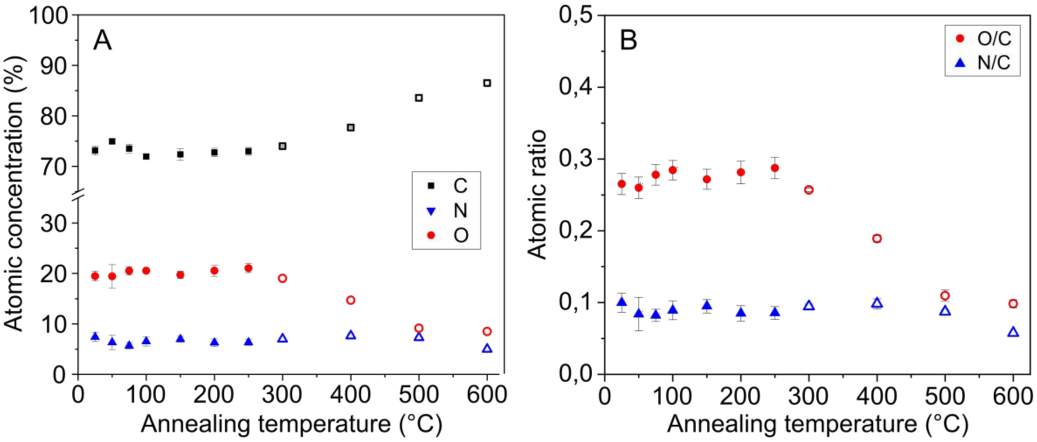

Figure S6. XPS determined atomic concentrations (A) and atomic ratios (B) of annealed PDA coatings on metal wires. The full markers correspond to annealing under a vacuum and the non-filled markers to annealing under an N_2 atmosphere.

SI-6. Polydopamine water absorption

Isothermal TGA measurements at 50 °C were performed on PDA powders, treated in three different conditions: 1) dried under vacuum at 25 °C, 2) dried under vacuum at 50 °C and 3) dried under vacuum at 50 °C and then left in ambient atmospheric conditions for three days. The isothermal curves are shown in Figure S7A and the equilibrium mass loss (EML) in Figure S7B. The results show a ~9 % EML in PDA powders dried under vacuum at 25 °C, while after drying under vacuum at 50 °C, the EML reduced to ~3%. For the PDA powders the were dried at 50 °C and then left in ambient conditions, the EML value was ~9%. This clearly shows that the observed mass loss is fully reversible between drying steps and exposure to atmospheric air. Thus in combination with the intense water peak that appears between 40° and 120 °C in the MS-TGA spectrum shown in Figure S7C, we assume that the EML observed at 50 °C is predominantly accounted to surface adsorbed water.

From the MS-TGA spectrum, it is also clear that the main outcome of thermal treatments of PDA is dehydration. However, it is impossible to distinguish whether at temperatures above 120 °C this is the removal of "strongly" bound water[2] or the product of dehydration reactions. CO_2 is also emitted by increasing temperature, however, the amounts become significant only above 250 °C.

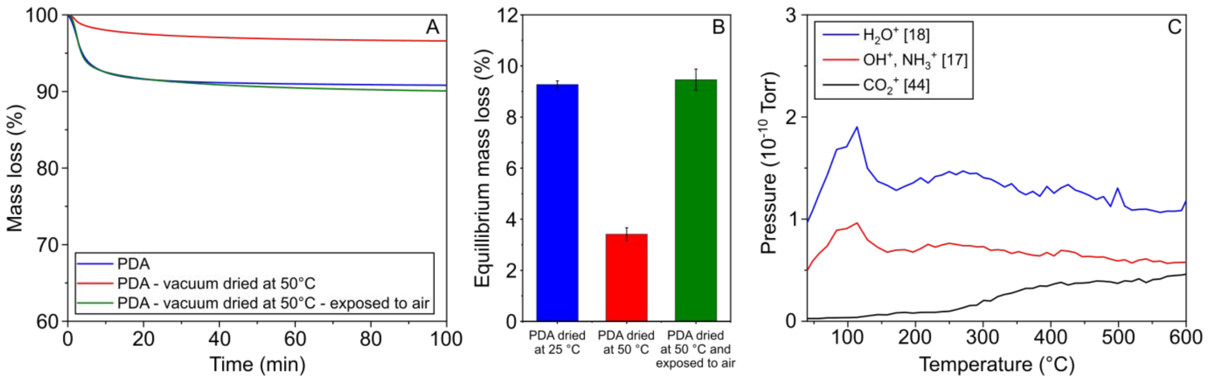

Figure S7. Isothermal TGA plots at 50 °C of PDA powders dried at 25 °C, dried at 50 °C and dried at 50 °C and then left in ambient conditions for 3 days (left). Average equilibrium mass loss values of the curves shown in A (B). MS-TGA plot of PDA powders (C).

SI-7. Polydopamine isothermal TGA curves, ^{13}C solid state NMR and FTIR curves.

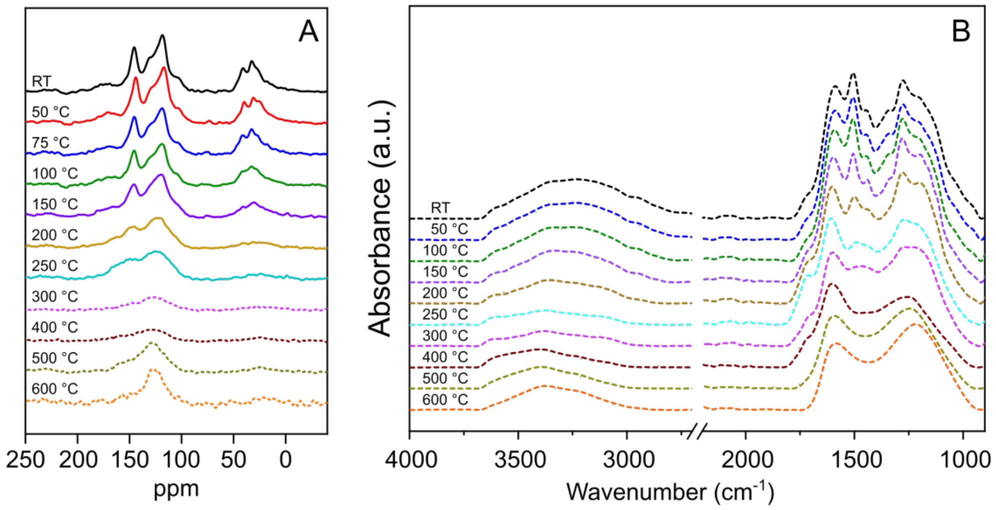

Figure S8. ^{13}C solid state NMR spectra obtained from PDA powders (A) and FTIR spectra obtained from PDA powders (B). In A and B, the solid lines correspond to PDA powders annealed under vacuum, while the dashed lines correspond to the resulting powders after the isothermal TGA analysis. In C, the intensity of the spectra obtained from powders annealed from 300 °C to 600 °C has been reduced (multiplier < 1) to facilitate comparison.

SI-8. Polydopamine coatings and powders FTIR spectra

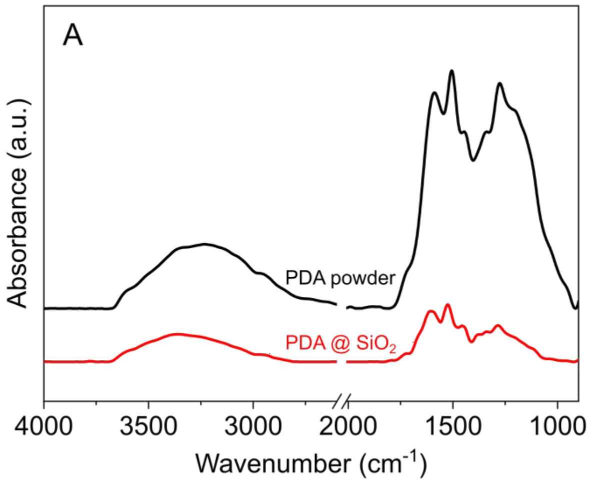

Figure S9. FTIR spectrum obtained from PDA powder and PDA film deposited on SiO_2 wafer (A). A representative sample of the PDA-coated SiO_2 wafer as well as a cross-sectional SEM image of the PDA coating on the surface of the SiO_2 wafer is shown in Figure S5A.

References

(1) Comini, N.; Huthwelker, T.; Diulus, J. T.; Osterwalder, J.; Novotny, Z. Factors Influencing Surface Carbon Contamination in Ambient-Pressure x-Ray Photoelectron Spectroscopy Experiments. *J. Vac. Sci. Technol. A* **2021**, *39* (4), 043203.

(2) Jastrzebska, M. M.; Isotalo, H.; Paloheimo, J.; Stubb, H. Electrical Conductivity of Synthetic DOPA-Melanin Polymer for Different Hydration States and Temperatures. *J. Biomater. Sci. Polym. Ed.* **1995**, *7* (7), 577–586.
